# 5-Bromo-2,7-dimethyl-3-(4-methyl­phenyl­sulfon­yl)-1-benzo­furan

**DOI:** 10.1107/S1600536814007181

**Published:** 2014-04-05

**Authors:** Hong Dae Choi, Pil Ja Seo, Uk Lee

**Affiliations:** a Department of Chemistry, Dongeui University, San 24 Kaya-dong, Busanjin-gu, Busan 614-714, Republic of Korea; b Department of Chemistry, Pukyong National University, 599-1 Daeyeon 3-dong, Nam-gu, Busan 608-737, Republic of Korea

## Abstract

In the title compound, C_17_H_15_BrO_3_S, the dihedral angle between the mean planes of the benzo­furan and 4-methyl­phenyl rings is 76.43 (5)°. In the crystal, mol­ecules are linked *via* pairs of C—H⋯O hydrogen bonds into inversion dimers that are further linked by Br⋯Br [3.6517 (4) Å] contacts about inversion centers into supra­molecular sheets that lie parallel to (111).

## Related literature   

For background information and the crystal structures of related compounds, see: Choi *et al.* (2011 ▶, 2012 ▶, 2013 ▶).
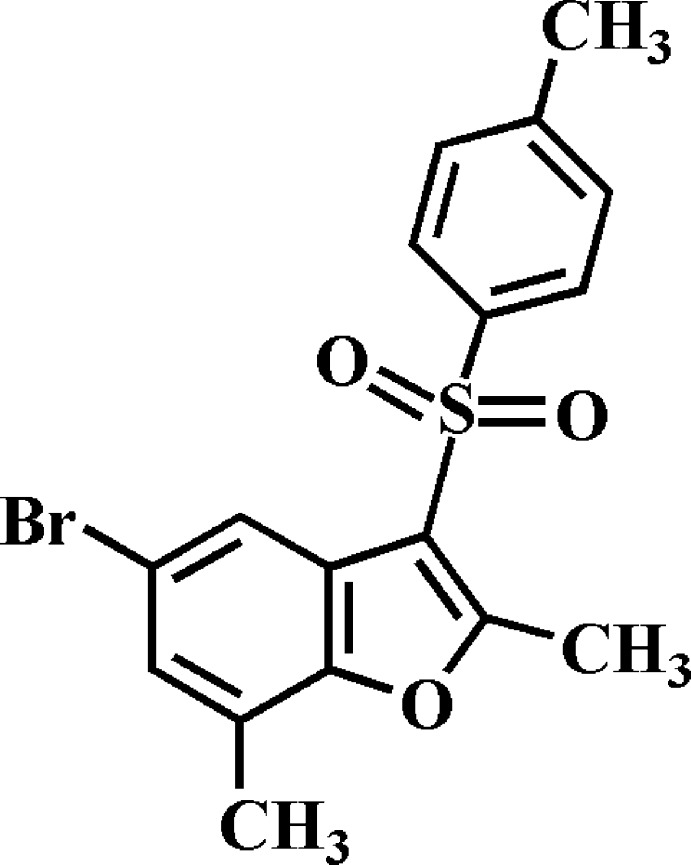


## Experimental   

### 

#### Crystal data  

C_17_H_15_BrO_3_S
*M* *_r_* = 379.26
Triclinic, 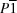

*a* = 8.1554 (2) Å
*b* = 9.9790 (2) Å
*c* = 10.1260 (2) Å
α = 77.410 (1)°
β = 77.114 (1)°
γ = 76.009 (1)°
*V* = 767.68 (3) Å^3^
*Z* = 2
Mo *K*α radiation
μ = 2.82 mm^−1^
*T* = 173 K
0.34 × 0.32 × 0.23 mm

#### Data collection  

Bruker SMART APEXII CCD diffractometer
Absorption correction: multi-scan (*SADABS*; Bruker, 2009 ▶) *T*_min_ = 0.543, *T*_max_ = 0.746
14016 measured reflections
3812 independent reflections
3336 reflections with *I* > 2σ(*I*)
*R*_int_ = 0.033

#### Refinement  

*R*[*F* ^2^ > 2σ(*F* ^2^)] = 0.028
*wR*(*F* ^2^) = 0.073
*S* = 1.04
3812 reflections
202 parameters
H-atom parameters constrained
Δρ_max_ = 0.36 e Å^−3^
Δρ_min_ = −0.42 e Å^−3^

### 

Data collection: *APEX2* (Bruker, 2009 ▶); cell refinement: *SAINT* (Bruker, 2009 ▶); data reduction: *SAINT*; program(s) used to solve structure: *SHELXS97* (Sheldrick, 2008 ▶); program(s) used to refine structure: *SHELXL97* (Sheldrick, 2008 ▶); molecular graphics: *ORTEP-3 for Windows* (Farrugia, 2012 ▶) and *DIAMOND* (Brandenburg, 1998 ▶); software used to prepare material for publication: *SHELXL97*.

## Supplementary Material

Crystal structure: contains datablock(s) I. DOI: 10.1107/S1600536814007181/gg2139sup1.cif

Structure factors: contains datablock(s) I. DOI: 10.1107/S1600536814007181/gg2139Isup2.hkl

Supporting information file. DOI: 10.1107/S1600536814007181/gg2139Isup3.cml

CCDC reference: 994776

Additional supporting information:  crystallographic information; 3D view; checkCIF report

## Figures and Tables

**Table 1:** Hydrogen-bond geometry (Å, °)

*D*—H⋯*A*	*D*—H	H⋯*A*	*D*⋯*A*	*D*—H⋯*A*
C13—H13⋯O2^i^	0.95	2.54	3.330 (2)	140

Symmetry code: (i) 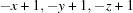.

**Fig. 1.**
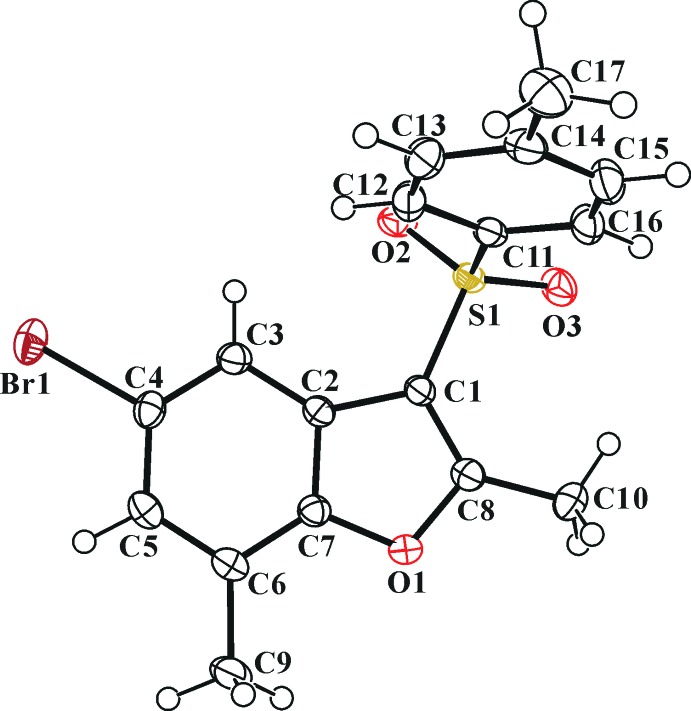
The molecular structure of the title molecule with the atom numbering scheme. The displacement ellipsoids are drawn at the 50% probability level. The hydrogen atoms are presented as small spheres of arbitrary radius.

**Fig. 2.**
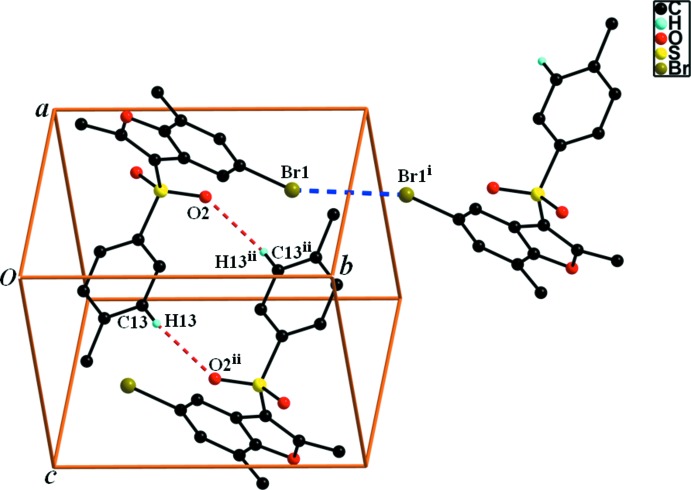
A view of the C—H···O and Br···Br interactions (dotted lines) in the crystal structure of the title compound. H atoms non-participating in hydrogen-bonding were omitted for clarity. [Symmetry codes: (i) - x + 1, - y + 2, - z; (ii) - x + 1, - y + 1, - z + 1]

## supplementary crystallographic information

### 1. Comment

As a part of our ongoing study of 5-bromo-2,7-dimethyl-1-benzofuran derivatives containing cyclohexylsulfonyl (Choi *et al.*, 2011), 4-fluorophenylsulfonyl (Choi *et al.*, 2012) and 4-methylphenylsulfinyl (Choi *et al.*, 2013) substituents in the 3-position, we report here on the crystal structure of the title compound.

In the title molecule (Fig. 1), the benzofuran ring system is essentially planar, with a mean deviation of 0.008 (1) Å from the least-squares plane defined by the nine constituent atoms. The 4-methylphenyl ring is essentially planar, with a mean deviation of 0.005 (1) Å from the least-squares plane defined by the six constituent atoms. The dihedral angle formed by the benzofuran ring system and the 4-methylphenyl ring is 76.43 (5)°. In the crystal structure (Fig. 2), molecules are linked *via* pairs of C—H···O hydrogen bonds (Table 1) into inversion dimers.

In the crystal, molecules are linked via pairs of C—H···O hydrogen bonds into inversion dimers that are further linked by C—H···O interactions and Br···Br [3.6517 (4) Å] contacts about inversion centers into supramolecular sheets that lie parallel with the (111) plane.

### 2. Experimental

3-Chloroperoxybenzoic acid (77%, 448 mg, 2.0 mmol) was added in small portions to a stirred solution of 5-bromo-2,7-dimethyl-3-(4-methylphenylsulfanyl)-1-benzofuran (312 mg, 0.9 mmol) in dichloromethane (40 mL) at 273 K. After being stirred at room temperature for 8h, the mixture was washed with saturated sodium bicarbonate solution and the organic layer was separated, dried over magnesium sulfate, filtered and concentrated at reduced pressure. The residue was purified by column chromatography (hexane-ethyl acetate, 4:1 *v*/*v*) to afford the title compound as a colorless solid [yield 69%, m.p. 474-475 K; *R*_f_ = 0.48 (hexane-ethyl acetate, 4:1 *v*/*v*)]. Single crystals suitable for X-ray diffraction were prepared by slow vaporation of a solution of the title compound in ethyl acetate at room temperature.

### 3. Refinement

All H atoms were positioned geometrically and refined using a riding model, with C—H = 0.95 Å for aryl and 0.98 Å for methyl H atoms, respectively. *U*_iso_ (H) = 1.2*U*_eq_ (C) for aryl and 1.5*U*_eq_ (C) for methyl H atoms. The positions of methyl hydrogens were optimized using the SHELXL97 command AFIX 137 (Sheldrick, 2008).

### Crystal data

**Table:**

C_17_H_15_BrO_3_S	*Z* = 2
*M**_r_* = 379.26	*F*(000) = 384
Triclinic, *P*1	*D*_x_ = 1.641 Mg m^−^^3^
Hall symbol: -P 1	Melting point = 475–474 K
*a* = 8.1554 (2) Å	Mo *K*α radiation, λ = 0.71073 Å
*b* = 9.9790 (2) Å	Cell parameters from 6793 reflections
*c* = 10.1260 (2) Å	θ = 2.7–28.3°
α = 77.410 (1)°	µ = 2.82 mm^−^^1^
β = 77.114 (1)°	*T* = 173 K
γ = 76.009 (1)°	Block, colourless
*V* = 767.68 (3) Å^3^	0.34 × 0.32 × 0.23 mm

### Data collection

**Table:**

Bruker SMART APEXII CCD diffractometer	3812 independent reflections
Radiation source: rotating anode	3336 reflections with *I* > 2σ(*I*)
Graphite multilayer monochromator	*R*_int_ = 0.033
Detector resolution: 10.0 pixels mm^-1^	θ_max_ = 28.4°, θ_min_ = 2.1°
φ and ω scans	*h* = −10→10
Absorption correction: multi-scan (*SADABS*; Bruker, 2009)	*k* = −13→13
*T*_min_ = 0.543, *T*_max_ = 0.746	*l* = −13→13
14016 measured reflections	

### Refinement

**Table:**

Refinement on *F*^2^	Primary atom site location: structure-invariant direct methods
Least-squares matrix: full	Secondary atom site location: difference Fourier map
*R*[*F*^2^ > 2σ(*F*^2^)] = 0.028	Hydrogen site location: difference Fourier map
*wR*(*F*^2^) = 0.073	H-atom parameters constrained
*S* = 1.04	*w* = 1/[σ^2^(*F*_o_^2^) + (0.0353*P*)^2^ + 0.3958*P*] where *P* = (*F*_o_^2^ + 2*F*_c_^2^)/3
3812 reflections	(Δ/σ)_max_ = 0.001
202 parameters	Δρ_max_ = 0.36 e Å^−^^3^
0 restraints	Δρ_min_ = −0.42 e Å^−^^3^

### Special details

**Table:**

Geometry. All esds (except the esd in the dihedral angle between two l.s. planes) are estimated using the full covariance matrix. The cell esds are taken into account individually in the estimation of esds in distances, angles and torsion angles; correlations between esds in cell parameters are only used when they are defined by crystal symmetry. An approximate (isotropic) treatment of cell esds is used for estimating esds involving l.s. planes.
Refinement. Refinement of F^2^ against ALL reflections. The weighted R-factor wR and goodness of fit S are based on F^2^, conventional R-factors R are based on F, with F set to zero for negative F^2^. The threshold expression of F^2^ > 2sigma(F^2^) is used only for calculating R-factors(gt) etc. and is not relevant to the choice of reflections for refinement. R-factors based on F^2^ are statistically about twice as large as those based on F, and R- factors based on ALL data will be even larger.

### Fractional atomic coordinates and isotropic or equivalent isotropic displacement parameters (Å^2^)

**Table:**

	*x*	*y*	*z*	*U*_iso_*/*U*_eq_	
Br1	0.50946 (3)	0.81656 (2)	−0.00546 (2)	0.03262 (8)	
S1	0.91173 (6)	0.31301 (5)	0.35042 (4)	0.02099 (10)	
O1	0.93317 (17)	0.24115 (13)	−0.01983 (13)	0.0222 (3)	
O2	0.88773 (18)	0.45707 (15)	0.36343 (14)	0.0274 (3)	
O3	1.06161 (18)	0.21433 (15)	0.38771 (14)	0.0287 (3)	
C1	0.9028 (2)	0.31299 (19)	0.18034 (17)	0.0199 (3)	
C2	0.8119 (2)	0.42615 (19)	0.09103 (18)	0.0195 (3)	
C3	0.7156 (2)	0.56082 (19)	0.10102 (19)	0.0225 (4)	
H3	0.6982	0.6000	0.1818	0.027*	
C4	0.6470 (2)	0.63398 (19)	−0.01324 (19)	0.0232 (4)	
C5	0.6705 (3)	0.5809 (2)	−0.13421 (19)	0.0251 (4)	
H5	0.6202	0.6369	−0.2094	0.030*	
C6	0.7667 (2)	0.4471 (2)	−0.14618 (18)	0.0230 (4)	
C7	0.8346 (2)	0.37528 (19)	−0.03066 (18)	0.0205 (3)	
C8	0.9720 (2)	0.2055 (2)	0.10962 (18)	0.0217 (4)	
C9	0.7931 (3)	0.3831 (2)	−0.2723 (2)	0.0308 (4)	
H9A	0.9106	0.3284	−0.2890	0.046*	
H9B	0.7745	0.4576	−0.3516	0.046*	
H9C	0.7114	0.3215	−0.2585	0.046*	
C10	1.0746 (3)	0.0626 (2)	0.1429 (2)	0.0284 (4)	
H10A	1.0974	0.0473	0.2366	0.043*	
H10B	1.1838	0.0523	0.0778	0.043*	
H10C	1.0108	−0.0065	0.1363	0.043*	
C11	0.7293 (2)	0.25019 (19)	0.44768 (18)	0.0206 (3)	
C12	0.5677 (3)	0.3361 (2)	0.4457 (2)	0.0270 (4)	
H12	0.5553	0.4269	0.3908	0.032*	
C13	0.4250 (3)	0.2879 (2)	0.5248 (2)	0.0295 (4)	
H13	0.3142	0.3461	0.5232	0.035*	
C14	0.4410 (3)	0.1561 (2)	0.60634 (19)	0.0267 (4)	
C15	0.6032 (3)	0.0716 (2)	0.6056 (2)	0.0303 (4)	
H15	0.6155	−0.0193	0.6604	0.036*	
C16	0.7476 (3)	0.1173 (2)	0.5263 (2)	0.0269 (4)	
H16	0.8580	0.0580	0.5260	0.032*	
C17	0.2839 (3)	0.1074 (3)	0.6937 (2)	0.0375 (5)	
H17A	0.3131	0.0064	0.7278	0.056*	
H17B	0.1937	0.1266	0.6384	0.056*	
H17C	0.2427	0.1575	0.7718	0.056*	

### Atomic displacement parameters (Å^2^)

**Table:**

	*U*^11^	*U*^22^	*U*^33^	*U*^12^	*U*^13^	*U*^23^
Br1	0.03337 (12)	0.01974 (11)	0.03988 (13)	−0.00075 (8)	−0.00655 (9)	−0.00013 (8)
S1	0.0221 (2)	0.0245 (2)	0.0167 (2)	−0.00315 (17)	−0.00529 (16)	−0.00419 (16)
O1	0.0252 (7)	0.0221 (6)	0.0191 (6)	−0.0024 (5)	−0.0035 (5)	−0.0065 (5)
O2	0.0345 (8)	0.0283 (7)	0.0229 (7)	−0.0090 (6)	−0.0053 (6)	−0.0089 (6)
O3	0.0226 (7)	0.0357 (8)	0.0258 (7)	−0.0006 (6)	−0.0085 (5)	−0.0031 (6)
C1	0.0201 (8)	0.0221 (9)	0.0162 (8)	−0.0025 (7)	−0.0034 (6)	−0.0026 (7)
C2	0.0199 (8)	0.0211 (8)	0.0173 (8)	−0.0053 (7)	−0.0027 (6)	−0.0021 (7)
C3	0.0254 (9)	0.0224 (9)	0.0199 (8)	−0.0060 (7)	−0.0024 (7)	−0.0042 (7)
C4	0.0231 (9)	0.0184 (8)	0.0257 (9)	−0.0044 (7)	−0.0026 (7)	−0.0004 (7)
C5	0.0277 (10)	0.0266 (10)	0.0209 (9)	−0.0087 (8)	−0.0067 (7)	0.0020 (7)
C6	0.0252 (9)	0.0272 (10)	0.0182 (8)	−0.0094 (8)	−0.0044 (7)	−0.0023 (7)
C7	0.0216 (8)	0.0207 (8)	0.0190 (8)	−0.0054 (7)	−0.0023 (7)	−0.0031 (7)
C8	0.0206 (9)	0.0248 (9)	0.0196 (8)	−0.0044 (7)	−0.0036 (7)	−0.0037 (7)
C9	0.0392 (12)	0.0361 (11)	0.0195 (9)	−0.0089 (9)	−0.0079 (8)	−0.0057 (8)
C10	0.0274 (10)	0.0236 (9)	0.0318 (10)	0.0015 (8)	−0.0057 (8)	−0.0072 (8)
C11	0.0222 (9)	0.0242 (9)	0.0154 (8)	−0.0031 (7)	−0.0039 (7)	−0.0045 (7)
C12	0.0278 (10)	0.0226 (9)	0.0269 (10)	−0.0009 (8)	−0.0055 (8)	−0.0007 (8)
C13	0.0227 (9)	0.0318 (11)	0.0301 (10)	0.0014 (8)	−0.0038 (8)	−0.0058 (8)
C14	0.0278 (10)	0.0326 (10)	0.0192 (9)	−0.0059 (8)	−0.0020 (7)	−0.0060 (8)
C15	0.0316 (11)	0.0264 (10)	0.0273 (10)	−0.0037 (8)	−0.0049 (8)	0.0041 (8)
C16	0.0235 (9)	0.0274 (10)	0.0252 (9)	0.0011 (8)	−0.0054 (8)	−0.0005 (8)
C17	0.0312 (11)	0.0460 (13)	0.0328 (11)	−0.0127 (10)	0.0004 (9)	−0.0028 (10)

### Geometric parameters (Å, º)

**Table:**

Br1—C4	1.9023 (19)	C9—H9A	0.9800
Br1—Br1^i^	3.6517 (4)	C9—H9B	0.9800
S1—O2	1.4343 (14)	C9—H9C	0.9800
S1—O3	1.4381 (14)	C10—H10A	0.9800
S1—C1	1.7400 (17)	C10—H10B	0.9800
S1—C11	1.7618 (19)	C10—H10C	0.9800
O1—C8	1.368 (2)	C11—C16	1.383 (3)
O1—C7	1.380 (2)	C11—C12	1.390 (3)
C1—C8	1.358 (3)	C12—C13	1.384 (3)
C1—C2	1.446 (2)	C12—H12	0.9500
C2—C7	1.391 (2)	C13—C14	1.385 (3)
C2—C3	1.395 (3)	C13—H13	0.9500
C3—C4	1.381 (3)	C14—C15	1.385 (3)
C3—H3	0.9500	C14—C17	1.507 (3)
C4—C5	1.395 (3)	C15—C16	1.385 (3)
C5—C6	1.391 (3)	C15—H15	0.9500
C5—H5	0.9500	C16—H16	0.9500
C6—C7	1.386 (3)	C17—H17A	0.9800
C6—C9	1.500 (3)	C17—H17B	0.9800
C8—C10	1.479 (3)	C17—H17C	0.9800
			
C4—Br1—Br1^i^	147.64 (6)	H9A—C9—H9B	109.5
O2—S1—O3	119.77 (9)	C6—C9—H9C	109.5
O2—S1—C1	106.40 (8)	H9A—C9—H9C	109.5
O3—S1—C1	109.32 (9)	H9B—C9—H9C	109.5
O2—S1—C11	107.59 (9)	C8—C10—H10A	109.5
O3—S1—C11	107.93 (9)	C8—C10—H10B	109.5
C1—S1—C11	104.85 (8)	H10A—C10—H10B	109.5
C8—O1—C7	107.07 (14)	C8—C10—H10C	109.5
C8—C1—C2	107.66 (15)	H10A—C10—H10C	109.5
C8—C1—S1	126.61 (14)	H10B—C10—H10C	109.5
C2—C1—S1	125.66 (14)	C16—C11—C12	120.53 (18)
C7—C2—C3	119.23 (17)	C16—C11—S1	120.14 (14)
C7—C2—C1	104.64 (16)	C12—C11—S1	119.33 (15)
C3—C2—C1	136.13 (16)	C13—C12—C11	119.24 (19)
C4—C3—C2	116.18 (17)	C13—C12—H12	120.4
C4—C3—H3	121.9	C11—C12—H12	120.4
C2—C3—H3	121.9	C12—C13—C14	121.07 (19)
C3—C4—C5	123.76 (18)	C12—C13—H13	119.5
C3—C4—Br1	118.52 (14)	C14—C13—H13	119.5
C5—C4—Br1	117.71 (14)	C13—C14—C15	118.72 (18)
C6—C5—C4	120.83 (18)	C13—C14—C17	119.98 (19)
C6—C5—H5	119.6	C15—C14—C17	121.30 (19)
C4—C5—H5	119.6	C16—C15—C14	121.17 (19)
C7—C6—C5	114.64 (17)	C16—C15—H15	119.4
C7—C6—C9	122.07 (18)	C14—C15—H15	119.4
C5—C6—C9	123.28 (17)	C11—C16—C15	119.25 (18)
O1—C7—C6	124.36 (16)	C11—C16—H16	120.4
O1—C7—C2	110.27 (15)	C15—C16—H16	120.4
C6—C7—C2	125.36 (18)	C14—C17—H17A	109.5
C1—C8—O1	110.36 (16)	C14—C17—H17B	109.5
C1—C8—C10	134.25 (17)	H17A—C17—H17B	109.5
O1—C8—C10	115.38 (16)	C14—C17—H17C	109.5
C6—C9—H9A	109.5	H17A—C17—H17C	109.5
C6—C9—H9B	109.5	H17B—C17—H17C	109.5
			
O2—S1—C1—C8	156.87 (17)	C3—C2—C7—O1	179.91 (15)
O3—S1—C1—C8	26.20 (19)	C1—C2—C7—O1	−0.49 (19)
C11—S1—C1—C8	−89.30 (18)	C3—C2—C7—C6	−0.9 (3)
O2—S1—C1—C2	−26.54 (18)	C1—C2—C7—C6	178.71 (17)
O3—S1—C1—C2	−157.20 (15)	C2—C1—C8—O1	0.3 (2)
C11—S1—C1—C2	87.29 (17)	S1—C1—C8—O1	177.42 (13)
C8—C1—C2—C7	0.1 (2)	C2—C1—C8—C10	−178.7 (2)
S1—C1—C2—C7	−177.03 (14)	S1—C1—C8—C10	−1.6 (3)
C8—C1—C2—C3	179.6 (2)	C7—O1—C8—C1	−0.6 (2)
S1—C1—C2—C3	2.5 (3)	C7—O1—C8—C10	178.59 (16)
C7—C2—C3—C4	0.8 (3)	O2—S1—C11—C16	−139.02 (15)
C1—C2—C3—C4	−178.64 (19)	O3—S1—C11—C16	−8.47 (18)
C2—C3—C4—C5	−0.5 (3)	C1—S1—C11—C16	107.99 (16)
C2—C3—C4—Br1	178.49 (13)	O2—S1—C11—C12	40.21 (17)
Br1^i^—Br1—C4—C3	70.15 (19)	O3—S1—C11—C12	170.75 (15)
Br1^i^—Br1—C4—C5	−110.76 (15)	C1—S1—C11—C12	−72.78 (16)
C3—C4—C5—C6	0.3 (3)	C16—C11—C12—C13	0.8 (3)
Br1—C4—C5—C6	−178.75 (14)	S1—C11—C12—C13	−178.44 (16)
C4—C5—C6—C7	−0.3 (3)	C11—C12—C13—C14	0.5 (3)
C4—C5—C6—C9	178.51 (18)	C12—C13—C14—C15	−1.2 (3)
C8—O1—C7—C6	−178.52 (17)	C12—C13—C14—C17	178.7 (2)
C8—O1—C7—C2	0.69 (19)	C13—C14—C15—C16	0.6 (3)
C5—C6—C7—O1	179.69 (16)	C17—C14—C15—C16	−179.28 (19)
C9—C6—C7—O1	0.9 (3)	C12—C11—C16—C15	−1.3 (3)
C5—C6—C7—C2	0.6 (3)	S1—C11—C16—C15	177.89 (15)
C9—C6—C7—C2	−178.21 (18)	C14—C15—C16—C11	0.6 (3)

Symmetry code: (i) −*x*+1, −*y*+2, −*z*.

### Hydrogen-bond geometry (Å, º)

**Table:**

*D*—H···*A*	*D*—H	H···*A*	*D*···*A*	*D*—H···*A*
C13—H13···O2^ii^	0.95	2.54	3.330 (2)	140

Symmetry code: (ii) −*x*+1, −*y*+1, −*z*+1.
